# The impact of COVID-19 pandemic outbreak on education and mental health of Chinese children aged 7–15 years: an online survey

**DOI:** 10.1186/s12887-021-02550-1

**Published:** 2021-02-24

**Authors:** Zhongren Ma, Sakinah Idris, Yinxia Zhang, Liu Zewen, Amaad Wali, Yunpeng Ji, Qiuwei Pan, Zulqarnain Baloch

**Affiliations:** 1 China-Malaysia National Joint Laboratory, Biomedical Research Center, Northwest Minzu University, Lanzhou, China; 2 Department of Psychiatry, Faculty of Medicine, Universiti Teknologi MARA, Shah Alam, Selangor Malaysia; 3 Faculty of Life Science and Technology, Kunming University of Science and Technology, Kunming, 650500 Yunnan China; 4 Department of Gastroenterology and Hepatology, Erasmus MC-University Medical Center, Rotterdam, the Netherlands

**Keywords:** COVID-19, Mental health, Online education, Children, China

## Abstract

**Background:**

The emerging of psychological problems triggered by COVID-19 particularly in children have been extensively highlighted and emphasized, but original research in this respect is still lagging behind. Therefore, we designed this study to evaluate the impact of COVID-19 pandemic on mental health and the effectiveness and attitudes towards online education among Chinese children aged 7–15 years.

**Methods:**

A detailed questionnaire, comprising of 62 questions was designed and parents or caretakers of 7 to 15 years old children were invited to participate via WeChat, a multi-purpose messaging, social media and mobile payment app, which is widely used by the Chinese population. A total of 668 parents across different regions of China were included.

**Results:**

During COVID-19 pandemic, 20.7 and 7.2% children report experiencing post-traumatic stress disorder (PTSD) and depressive symptoms due to the COVID-19 pandemic. PTSD and SMFQ-P scores are significantly higher in middle school and boarding school students compared to primary and day school students. Multiple logistic regression analysis revealed that school system and province of origin are factors significantly associated with developing PSTD symptoms. 44.3% respondents feel online education is effective in gaining knowledge and improving practical and communications skills. 78.0% believe the online education system is efficient. Overall 79.8% respondents are satisfied and children can adapt to this new education system. During the COVID-19 pandemic, we found 1 in five children have PTSD and 1 in 14 children have depressive symptoms.

**Conclusion:**

In summary, COVID-19 epidemic has caused PTSD and depression symptoms among Chinese children aged 7 to 15 years. In general, a large proportion of respondents are satisfied with online education, but still a substantial proportion of students are not comfortable with this new form of learning. Authorities should optimize online education systems and implement effective interventions to cope with the psychological effects of COVID-19 on children, as it is affecting the global population and remains uncertain when it will end.

## Backgrounds

The consequences of the ongoing coronavirus disease 2019 (COVID-19) pandemic is infiltrating in every aspects of our society and daily lives. However, what is the effectiveness and draw-backs of these control measures and how the pandemic will eventually remain largely uncertain. According to United Nation estimates, 188 countries have enforced nationwide school closures, resulting in more than 90% enrolled students are out of school and confined at home [1]. School closure, living in isolation, and fearing risk of the infection are expected to have major impact on education, psychological health, and well-being of children [1]. Epidemiological evidence suggests that approximately 5–12% of people may develop post-traumatic stress disorder (PTSD) after a traumatic event [2]. The emerging of psychological problems triggered by COVID-19 particularly in children have been extensively highlighted and emphasized, [3, 4] but original research in this respect is still lagging behind.

In response to the COVID-19 outbreak, the Chinese Government was the first to order nationwide school closure as an emergency measure to minimize the risk of spreading [4, 5]. The Ministry of Education estimates that more than 220 million children and adolescents are confined to their homes. This includes 180 million primary and secondary students and 47 million preschool children [4]. To minimize educational losses, immense efforts were made from different stake holders to create online courses and deliver them through electronic media and internet [5]. As China has experienced the first wave of the epidemic and the children were confined at home for the longest period two or more months, it is an ideal setting for comprehensively evaluating the impact of COVID-19 pandemic controlling measures on education and mental health of the children. Current study was design to assess the effects and attitudes towards online education, the prevalence of PTSD and depression symptoms, and their associated factors among children aged 7 to 15 years.

## Methods

We conducted a cross-sectional survey from April 11th to 17th, 2020. The survey involved a questionnaire that was distributed through online version via WeChat, a Chinese multi-purpose messaging, social media and mobile payment app. It is widely used in China with monthly active users estimated of one billion. Parents or caretakers of 7 to 15 years old children were invited to participate in this study. They are requested to involve their children to complete the survey together.

This study uses purposive sampling. A form containing the study questionnaire was distributed among specific social media groups comprising parents who have at least a child age between 7 and 15 years old and who also follow the online education. Messages were sent to them to ensure the appropriate selection of study participants. A friendly reminder was sent to potential respondents to ensure the highest possible response rate. Participants were not aware of the study aim or outcomes to reduce the risk of any possible bias. The questionnaire was self-administered without intervention by the authors or any specific person, and it did not contain any identifying data of the participants to ensure confidentiality. Questionnaires with incomplete information or missing data were excluded from the analysis.

### Survey instrument

A detailed questionnaire, comprising of 62 questions was designed in both English and Chinese languages, and the Chinese version was used in this study. The questionnaire comprised of 15 basic demographic characteristics, such as children’s gender, age, their education as well as data about their parent’s marital status, level of education, profession, and income. The questionnaire also addressed the usability for online education of the children in three aspects; (1) the effectiveness, means the accuracy and completeness in which the child get the knowledge (3 questions), (2) the efficiency, to measure through task/homework completion (3 questions), and (3) satisfaction, means the comfort and acceptability of online education (2 questions). Each question comprises of five options ranging from 1-not at all, 2- a little bit, 3-moderately, 4-quite a bit, and 5 -extremely. Several questions related to the online education is our primary outcome.

Additionally, the survey also included two mental health assessments that measure level of anxiety and depression which is our outcome variables.

The psychological impact was evaluated using a validated scale, i.e. Impact of Events Scale-Revised (IES-R) [6, 7]. IES is a self-reporting measure used to assess the response to a specific stressful life event [8]. It is composed of 22 items, each with a Likert rating scale from 0 to 4 (0 not at all; 1 a little bit; 2 moderately, 3 quite a bit; 4 extremely). The maximum score is 88. A higher score indicates a greater concern for PTSD. IES-R has been translated in Chinese. The reliability and validity have been extensively demonstrated [9]. It is frequently used in trauma research worldwide [10, 11].

The SMFQ-P [12] is a 13-item shortened version of the 33-item MFQ [13]. It was developed in response to the need for a brief depression measure to reduce participant burden in research trials, while still retaining strong criterion validity [12]. Responses are rated on a 3-point scale (0 = not true, 1 = sometimes, and 2 = true). The SMFQ-P has matched versions completed by the parent (SMFQ-P) and is freely available [13]. The SMFQ-P takes approximately 3 to 5 min to complete and has been validated with clinical [14] and nonclinical [12, 15] samples. A score 12 or above was commonly used to indicate clinically significant depression. The MFQ was originally designed for use with children. It is based on DSM-III-R (stands for Diagnostic and Statistical Manual of Mental Disorders) symptom criteria and has been recommended by National Institute for Health and Clinical Excellence (2005) for the screening of depression in children and adolescents.

### Statistical analysis

Descriptive and inferential statistics were calculated using SPSS version 20.0 for Windows (SPSS Inc., Chicago IL). Descriptive statistics analysis was done to demonstrate the demographic characteristics of the participants. A univariate analysis was first examined to screen statistically significant variables to be included in multivariate logistic regression analyses. The estimations of the strengths of associations were revealed by the odds ratio (OR) with a 95% confidence interval (CI). All statistical tests were two-sided and a *p-*value < 0.05 was considered as statistically significant. We performed all statistical analyses using (IBM) SPSS version 22.0.

### Ethical approval

The protocol used in this study was in accordance with the Declaration of Helsinki and was approved by the Ethics Committee at Northwest Minzu University Lanzhou, China. Informed consent was individually obtained from the parents.

## Results

In total, 680 individuals from 27 provinces, autonomous regions and municipalities across China were invited to participate in the survey by filling the questionnaires. 12 declined to continue the survey, resulting in 668 participants with children aged 7–15 years finally included in the study. Detailed demographic characteristics of the children and their families are presented in Table 1. Majority participants are from Henan (34.6%), Gansu (25.0%), Inner Mongolia (10.8%), Liaoning (9.3%) and Zhejiang (8.1%) provinces and the remaining are from 22 other provinces, autonomous regions or municipalities across China. Majority participants are day school students (87.1%), and 95.5% lived with parents In terms of ethnicity, 87.7% are Hans and 12.3% are from other ethnicities. 75.4% live in urban areas and 24.6% have rural background (Table 1).

**Table 1:** Demographic characteristics of the respondents (*n* = 668)

Child Demographics			Parents Demographics		
Characteristics	Frequency	Percentage	Characteristics	Frequency	Percentage
Sex			**Marital status**		
Male	336	50.3%	Married	643	96.3
Female	332	49.7%	Divorced	25	3.7
Age (years)			**Number of child**		
7	53	7.9	Not filled	19	2.8
8	68	10.2	1	257	38.5
9	90	13.5	2	339	50.7
10	112	16.8	> 2	53	7.9
11	51	7.6	**Mother education**		
12	20	3.0	Not filled	24	3.6
13	50	7.5	Illiterate	8	1.2
14	91	13.6	Primary	42	6.3
15	133	19.9	Middle	176	26.3
School level			High school	98	14.7
Primary	394	59.0	Diploma	103	15.4
Middle	274	41.0	Undergraduate	142	21.3
School type			Master	49	7.3
Boarding school	86	12.9	PhD	26	3.9
Day school	582	87.1	**Father education**		
Children live with			Not filled	29	4.3
Parents	638	95.5	Illiterate	3	.4
Grandparents	25	3.7	Primary	26	3.9
Other caregivers	5	.7	Middle	192	28.7
Ethnicity			High school	100	15.0
Han	586	87.7	Diploma	85	12.7
Other ethnic	82	12.3	Undergraduate	161	24.1
Residence			Master	44	6.6
Rural area	504	75.4	PhD	28	4.2
Urban area	164	24.6	**Mother profession**		
Monthly Income (RMB)			Not filled	26	3.89
< 3000	80	12.0	Farmer	81	12.12
3001–5000	173	25.9	Teacher	94	14.07
5001–10,000	225	33.7	Nurse	13	1.95
> 10,000	161	24.1	Doctor	23	3.44
Not filled	29	4.3	Civil servant	34	5.1
Province			Private sector	118	17.66
Gansu	167	25.0	Businessman	32	4.09
Henan	231	34.6	Others	247	36.98
Liaoning	62	9.3	**Father profession**		
Inner Mongolia	72	10.8	Not filled	27	4.0
Zhejiang	54	8.1	Farmer	73	10.9
Others	82	12.27	Teacher	51	7.6
			Doctor	19	2.8
			Civil Servant	59	8.8
			Private sector	133	19.9
			Businessman	55	8.2
			Others	251	37.6

562 out of 668 respondents have reported that their families are well-aware of COVID-19. Overall, respondents think their children are affected psychologically (46.7%), emotionally (35.8%), socially (34.0%) and physically (27.4%) by the COVID-19 pandemic. In contrast, 37.4% think their children are not affected at all. 73.2% participants reported that their children have returned to normal routine after the control of COVID-19 in China (**Appendix****Table 6**). Interestingly, 138 participants reported that their children are affected both physically and psychologically, and 67 reported to be affected physically, psychologically, socially and emotionally.

In this study, an IES-R score of > 20 was used to estimate the prevalence of PTSD, and 20.66% children have the score above 20 **(**Table 2**)**. PTSD symptoms were significantly more prevalent in middle school (*p* = 0.05) and boarding school students (*p* = 0.004) compared to primary school and day school students, respectively. SMFQ-P score of > 12 was used to estimate the prevalence of depression among children. 7.18% students have the score above 12. SMFQ-P based depression was also significantly more prevalent in middle school (*p* = 0.032) and boarding school students (*p* = 0.02) compared to primary and day school students, respectively (Table 2).

**Table 2:** Prevalence of PSTD (IES-R) and depression (SMFQ-P) symptoms among children aged 7 to 15 years

	Total	PSTD (%)	P-value	SMFQ-P (%)	P-value
**Overall prevalence**	668	20.7		7.2	
**Education level**			**0.05**		**0.032**
Primary	394	71 (18.0)		21 (5.3)	
Middle	274	67 (24.4)		27 (9.8)	
**School type**			**0.004**		**0.02**
Boarding school	86	28 (32.6)		12 (13.9)	
Day school	582	110 (18.9)		36 (6.2)	
**Live with**			0.058		0.18
Parents	638	128 (20.1)		44 (6.9)	
Grandparents	25	7 (28.0)		4 (16.0)	
Caregivers	5	3 (60.0)		0	
**Province**			**0.003**		0.44
Others	82	23 (28.0)		9 (11.0)	
Gansu	167	29 (17.4)		14 (8.4)	
Henan	231	61 (26.41		17 (7.4)	
Inner Mongolia	62	9 (14.5)		3 (4.9)	
Liaoning	72	13 (18.0)		3 (4.2)	
Zhejiang	54	3 (5.5)		2 (3.7)	

PSTD: Post-traumatic stress disorder, SMFQ-P, Short Mood and Feeling questionnaire

44.3% respondents feel online education is effective (scored as average, good or excellent), in terms of gaining knowledge and improving practical and communications skills. 78.0% think it is efficient that students can complete the assigned tasks, communicate with teachers and take advantages of the multimedia format. 78.0% responded that they are overall satisfied and children can adapt to the online education system (Table 3). Correlation analysis revealed that children age (*p*-value< 0.0001), school level (p-value< 0.0001), provincial (*p* = 0.006) l and residential background (*p* = 007), and family income status (*p* = 0.008) is significantly linked with the effectiveness and satisfaction of the online education system (Table 4). The total IES-R scores are significantly correlated with type of school (p = 0.006), children living with whom (*p* = 0.044), and provincial background (*p* = 0.016). The SMFQ-P scores are significantly correlated with child age (*p* = 0.004), school level (*p* = 0.10), school type (*p* = 0.001), provincial back ground (p = 0.016) and area of residence (*p* = 0.032) (Table 4).

**Table 3:** Experience and attitudes towards online education

Variables	No	Less	Average	Good	Excellent
Effective	51 (7.6)	321 (48.0)	211 (31.6)	65 (9.73)	20 (3.0)
Efficient	8 (1.9)	139 (20.1)	303 (45.4)	166 (24.85)	52 (7.8)
Satisfied	23 (3.4)	112 (16.8)	318 (47.6)	142 (21.26)	73 (10.9)

**Table 4:** Correlation between online education variables, IES-R, MFQ-P and demographic characteristics

		Sex	Age	School level	School type	Child live with whom	Province	Ethnicity	Residence	Income/M
**Effectiveness**	Pearson r	.041	.149^**^	.172^**^	−.037	.007	.040	−.071	−.105^**^	−.004
	Sig (2-tailed)	.295	**.000**	**.000**	.338	.857	.302	.065	**.007**	.923
**Efficiency**	Pearson r	.055	.202^**^	.229^**^	−.027	.047	.106^**^	−.047	−.113^**^	−.022
	Sig (2-tailed)	.156	**.000**	**.000**	.483	.223	**.006**	.223	**.004**	.566
**Satisfaction**	Pearson r	.030	.173^**^	.166^**^	−.045	.032	.078^*^	−.077^*^	−.037	−.102^**^
	Sig (2-tailed)	.440	**.000**	**.000**	.247	.410	**.043**	**.047**	.339	**.008**
**IES-R-P total**	Pearson r	−.022	.047	.052	−.106^**^	.078^*^	−.093^*^	−.028	.100^**^	−.101^**^
	Sig (2-tailed)	.576	.221	.179	**.006**	**.044**	**.016**	.477	.009	.009
**SMFQ-P score**	Pearson r	−.029	.111^**^	.100^*^	−.130^**^	.057	−.093^*^	−.013	.083^*^	−.058
	Sig (2-tailed)	.459	**.004**	**.010**	**.001**	.141	**.016**	.729	**.032**	.136

IES-R, Impact of Event Scale-Revised, MFQ-P, Mood and Feeling questionnaire-parents *p < 0.05

According to univariate analysis, children education level (primary or middle school), school system (day or boarding school), living status, provincial background and father profession are significantly linked with PSTD symptoms. All these significant factors were further included into multiple logistic regression analysis. School system (*p* = 0.05) and provincial background (*p* = 0.013) remain as the significant factors for developing PSTD symptoms in children (Table 5).

**Table 5:** Logistic regression analysis of factors accosted with the prevalence of PSTD symptoms

Variables	Numbers	Sig.	OD(95% CI)
School level		.097	
Primary	394		1
Middle	274		1.50 (0.93–2.41)
School type		0.05	
Boarding school	86		1
Day school	582		0.56 (0.31–1.02)
Children Live with		0.43	
Parents	638		1
Grand parents	25		1.41 (0.56–3.55)
Caregivers	5		3.03 (0.41–22.65)
Province		0.013	
Others	82		1
Gansu	167		0.48 (0.24–0.94)
Henan	231		0.63 (0.33–1.22)
Inner Mongolia	62		0.27 (0.10–0.72)
Liaoning	72		0.74 (0.33–1.64)
Zhejiang	54		0.15 (0.042–0.55)
Father Profession			
Not fill	27		1
Farmer	73		1.45 (0.53–4.08)
Teacher	51		0.78 (0.24–2.50)
Doctor	19		1.2 (0.29–4.97)
Civil servant	59		0.54 (0.16–1.78)
Private sector	133		0.64 (0.23–1.78)
Businessman	55		0.31 (0.09–1.09)
Others	251		0.72 (0.28–1.86)

OD: Odd ratio, CI: Confidence interval

Variable(s) entered on step 1: school level, school type, child living with whom, province, father profession

Univariate analysis revealed that school level (primary or middle school) and system (day or boarding school) are significantly associated with depression among children. However, both factors are not significant in multivariate logistic analysis (**Appendix****Table 7**).

## Discussion

Implementations of public health emergency control measures have major impacts on the psychology of young children [3, 4]. The negative consequences of COVID-19 pandemic is penetrating every aspects of the society, not only health but also economy, education, religion and politics [16, 17]. Negative psychological effects are expected to happen and exacerbate when children are confined to their homes in the absence of outdoor activities and interpersonal communication with same aged friends [17–19]. It would be very common that they are feeling anxious, isolated and disappointed. In this study, we first quantified the effects of COVID-19 on mental health of children aged 7 to 15 years in China. We further identified important factors associated with the development of PTSD and depression symptoms. We found that 20.7 and 7.16% children experienced PSTD and depression symptoms due to the COVID-19 pandemic. The high prevalence rate of psychological symptoms in children is not surprising given the unprecedented negative effects of COVID-19 on everyone’s daily lives. Children are confined at home, worrying about their education, and fearing the risk of contracting infection, and their families may suffer from substantial economic losses [3, 4, 20, 21]. Furthermore, the overwhelmed news reports and sensational news headlines related to COVID-19 have inevitably triggered worry, fear and anxiety among the general population including children [3, 21]. These factors collectively and may synergistically affect the mental health of children.

PTSD and SMFQ-P scores are significantly higher in middle school and boarding school students compared to primary and day school students. Significantly higher PSTD and SMFQ-P scores in middle school students is probably related to their better awareness towards COVID-19, as SARS-CoV-2 is highly contagious [22]. Furthermore, they are more likely concerned about their studies and future plans [4, 23, 24]. In contrast, primary school children are not yet mature in childhood; therefore they are naturally less worried. Day school students and students living with parents have lower PSTD and SMFQ-P scores. This is consistent with previous studies that children living in institutions [25], children not living with parents, and orphans [26, 27] have higher risk developing psychological disorders.

In response to COVID-19, the Chinese government took strict measures; including school closure, travel ban, lockdown, and within-population quarantine. The Chinese New Year national holidays were extended but family visiting and social gathering were strictly banned [28]. Although control measures are universally implemented, the levels of intensity could still vary among different provinces, and between rural and urban areas. This may partially explain why students from different provinces and different resident areas have different degrees of psychological impact.

In response to school closure, the education sector has to suddenly switch to online learning. However, online education requires systematic thinking, design and implementation. But at this urgent circumstance especially China as the first epicenter, there was no sophisticated preparation, and the format of e-learning could vary dramatically among different regions and schools. Therefore, we could only perform a general assessment. Overall, a large proportion of respondents are stratified with online education. Children age, school level, provincial and residential background, and family income status are significantly associated with the effectiveness and satisfaction of the online education system. This can be explained by two main reasons. Children with old age are technically more capable of adapting to this new form of education. Families or regions with higher socioeconomic status are more accessible to high technologies and digital devices. Thus, there are still a substantial proportion of students who are not comfortable with online education and are not participating effectively. The education sectors and other stakeholders should pay attention to optimize the education program and ensure equal access to the technology and tools. Effective and universally accessible online education will not only minimize academic loss, but also mitigate the mental health of children by actively involving teachers, parents and friends.

Of note, there are some limitations in this study. First, the survey was invited via the WeChat app based on social network. This may lead to bias, but our respondents are widely distributed across different parts of China with different background. Second, due to the nature of online survey, we could only use self-reporting instead of diagnostic interview for assessing psychiatric morbidity. Last but not the least; we could not study the content of online education in detailed, as the format varies dramatically among different regions and schools in China.

## Conclusion

In summary, we found that 20.7 and 7.16% of children aged 7 to 15 years in China experienced psychological issues due to the COVID-19 epidemic. These symptoms are more prevalent in middle school and boarding school students compared to primary and day school students. In general, a large proportion of respondents are satisfied with online education, but still a substantial proportion of students are not comfortable with this new form of learning. Therefore, authorities should continuously optimize the online education program and ensure equal access to digital learning opportunities.

## Data Availability

The datasets generated and analyzed during the current study are not publicly available due to the risk of compromising the individual privacy of participants, but are available from the corresponding author on reasonable request.

## Appendix 1

**Table 6:** Responses to different questions related to knowledge and overall effects of COVID-19 (n=668)

	Yes	Percentage
Is your family well-aware of COVID-19?	562	84.1
Are your child back to routine after the control of COVID-19 epidemic?	489	73.2
COVID-19 affected your child physically	183	27.4
COVID-19 affected your child psychologically	312	46.7
COVID-19 affected your child emotionally	239	35.8
COVID-19 affected your child socially	227	34
COVID-19 did not affected your child	250	37.4

**Table 7:** Logistic regression analysis on factors associated with the prevalence of depressive symptoms.

	Numbers	Sig.	OD( 95% CI)
School level		0.16	
Primary	394		1
Middle	274		1.59(0.83-3.05)
School type		0.09	
Boarding school	86		1
Day school	582		0.51(0.24-1.11)

OR: odd ratio, CI: Confidence Interval

## Abbreviations

PTSD: Post-traumatic stress disorder
COVID-19: Coronavirus disease 2019
SMFQ-O: Mood and Feeling questionnaire-parents
IES-R: Impact of Events Scale-Revised
OR: Odds ratio
CI: Confidence interval
